# Current Practices in Vestibular Migraine Management Among Canadian Otolaryngologists: A National Survey

**DOI:** 10.3390/audiolres16030082

**Published:** 2026-05-27

**Authors:** Raisa Chowdhury, Angelina Tohme, Daniel Lelli, Darren Tse

**Affiliations:** 1 Faculty of Medicine and Health Sciences, McGill University, Montreal, QC H3A 0G4, Canada; 2 Department of Otolaryngology-Head & Neck Surgery, The Ottawa Hospital, Ottawa, ON K1H 8L6, Canada; 3 Clinical Epidemiology, The Ottawa Hospital Research Institute, Ottawa, ON K1H 8L6, Canada; 4 Department of Medicine, Division of Neurology, The Ottawa Hospital, Ottawa, ON K1H 8L6, Canada

**Keywords:** vestibular migraine, migraine, dizziness, vertigo, otolaryngology, neuro-otology, survey study, medical education, clinical practice guidelines, vestibular rehabilitation

## Abstract

Background/Objectives: Vestibular migraine is a common but often underrecognized cause of dizziness in otolaryngology practice. Although awareness has increased, variation in clinician training and management may contribute to inconsistent care. This study evaluated current diagnostic and treatment practices of Canadian otolaryngologists for vestibular migraine, including familiarity with diagnostic criteria, therapeutic approaches, perceived barriers, and educational needs. Methods: A national cross-sectional electronic survey was distributed to practicing and emeritus members of the Canadian Society of Otolaryngology–Head and Neck Surgery from February to April 2025. The 14-item survey assessed demographics, clinical exposure to dizzy patients, residency training, diagnostic familiarity, treatment patterns, referral practices, barriers to care, and preferred educational resources. Responses were anonymized and analyzed using descriptive statistics. Results: Forty-four otolaryngologists completed the survey (response rate: 7.4%). Most respondents reported being very familiar (59.1%) or moderately familiar (38.6%) with vestibular migraine diagnostic criteria, and 97.7% reported currently diagnosing and/or treating these patients. However, only 15.9% had received extensive residency training specific to migraine or vestibular migraine. Common treatments included lifestyle and dietary modification (90.9%), nutraceutical supplements (59.1%), tricyclic antidepressants (54.5%), and analgesics (52.3%). Vestibular rehabilitation therapy (29.5%) and calcitonin gene-related peptide-targeted therapies (<10%) were used less frequently. Major barriers were clinical time constraints (65.9%), lack of training or knowledge (54.5%), and diagnostic complexity (47.7%). Clinical guidelines (70.5%) and continuing medical education courses (65.9%) were identified as the most valuable supports. Conclusions: Among surveyed Canadian otolaryngologists engaged in dizziness and vestibular migraine care, substantial heterogeneity existed in training and management practices. Standardized guidance, enhanced education, and interdisciplinary collaboration may improve consistency of care and patient outcomes.

## 1. Introduction

Vertigo and migraine have been correlated since the 19th century, yet their relation has only recently been subjected to thorough investigation [1,2,3]. Vestibular migraine (VM) is recognized as a common yet often underdiagnosed condition. VM occupies a unique diagnostic space at the intersection of headache medicine and vestibular disorders. Although diagnostic criteria have been proposed by the Bárány Society and included in the appendix of the International Classification of Headache Disorders, 3rd edition (ICHD-3), overlap remains between vestibular migraine, migraine with or without aura, Ménière’s disease, and other episodic vestibular syndromes. This diagnostic ambiguity contributes to variability in terminology, clinical interpretation, and management approaches across specialties. VM is estimated to affect 1% to 2.7% of the population and accounts for approximately 25% of patients presenting with dizziness to tertiary-level neuro-otology clinics [4,5,6]. Given its diagnostic complexity and symptom overlap with other vestibular disorders, it is likely this remains an underestimate.

In 2012, the Bárány Society and the International Headache Society jointly published diagnostic criteria for vestibular migraine, which were later updated in 2021 [7,8,9]. These developments reflect growing recognition and clinical interest in this distinct disorder.

Although VM is often managed using broader migraine-directed therapeutic strategies, the optimal treatment approach remains unclear. While there is growing evidence supporting shared strategies, definitive guidelines have yet to be established. As migraine management traditionally falls under the care of the neurologist, otolaryngologists may not feel adequately equipped to initiate treatment. However, patients with dizziness frequently present first to otolaryngology clinics, and the responsibility to recognize and manage VM often begins in this setting [10,11]. This highlights VM as a condition where neurology and otolaryngology intersect in both diagnosis and care. This study aims to provide insights into the current practice of otolaryngologists across Canada with regard to diagnosing and managing vestibular migraine.

## 2. Materials and Methods

Ethical approval for the study was obtained from the Ottawa Hospital Science Network Research Ethics Board (protocol ID 20240641-01H). The study and survey were also approved for distribution by the Canadian Society of Otolaryngology—Head and Neck Surgery (CSOHNS). A cross-sectional survey was sent out on 13 February 2025. The survey was disseminated electronically via email to all practicing and emeritus CSOHNS members, who comprised the study population. Residents, fellows, medical students, and non-otolaryngologist physicians were excluded. Incomplete survey responses were excluded from final analysis.

Participation was voluntary, and no incentives were offered, with confidentiality and anonymity maintained. Completion of the survey implied informed consent to participate. There was a further prompt to do the survey 1 month and 2 months later, and the survey was open for participation until 3 April 2025. There was no follow-up beyond initial participation.

The survey comprised 14 structured questions, including multiple-choice and Likert-scale items, with one open-ended question to capture qualitative feedback (Appendix A). The questionnaire was developed by the study team based on existing vestibular migraine literature, Canadian clinical practice considerations, and expert input from clinicians involved in neuro-otology and headache medicine. Questions were designed to assess familiarity with vestibular migraine diagnostic criteria, management approaches, barriers to care, and educational needs. The survey underwent internal review for clarity and content relevance prior to distribution; however, it was not formally validated or externally piloted. Topics included demographic and professional information (province/territory of practice, subspecialty focus, practice setting, years in practice), familiarity with diagnostic criteria, training received during residency, clinical practices related to dizzy patients, migraine management strategies, referral patterns, perceived barriers, and educational/resource needs. Participants were allowed to select multiple responses where appropriate, and one question allowed for optional free-text comments.

Data collection was carried out through a survey form online that remained open while the survey was active. Responses were anonymized to maintain confidentiality. Descriptive statistics were used to summarize the data, with categorical variables presented as frequencies and percentages. Given the exploratory and descriptive nature of this national survey study, a formal sample size calculation was not performed.

## 3. Results

The survey was distributed to 593 practicing and emeritus members of the CSOHNS. Email analytics showed that 493 recipients (83.1%) opened the invitation email and 169 (34.3%) clicked the survey link. A total of 44 otolaryngologists completed the survey, corresponding to an overall response rate of 7.4%. Ontario (29.5% of respondents) and Quebec (22.7% of respondents) had the largest participation rates, with additional participants from British Columbia, Alberta, Nova Scotia, and other provinces (Table 1). Most respondents identified their primary practice focus as either otology/neurotology (45.5%) or general otolaryngology (40.9%). Most respondents worked at academic teaching hospitals (45.5%) or community-based practices (31.8%). Practice years varied from less than five years to more than twenty-one years.

The majority of respondents were either very familiar (59.1%) or moderately familiar (38.6%) with the published VM diagnostic criteria. Regarding formal training, only 15.9% of respondents reported extensive training specific to migraine and VM during residency, 45.5% of respondents reported receiving some training on VM in residency, and 38.6% of respondents stated no formal training at all.

Dizzy patients were common, with 81.8% of respondents encountering such patients multiple times per week. Around 11.4% saw them multiple times per month. Nearly all respondents (97.7%) reported that they currently diagnose and/or treat patients with VM.

Among those that treat VM, the most commonly used interventions were lifestyle and dietary modifications (90.9%), tricyclic antidepressants (54.5%), analgesics (52.3%), nutraceutical supplements (59.1%) and triptans (36.4%). Fewer participants reported using vestibular rehabilitation therapy (29.5%), selective serotonin reuptake inhibitors (SSRI) or serotonin-norepinephrine reuptake inhibitors (SNRI) (22.7%), beta blockers (18.2%), oral gepant calcitonin gene-related peptide (CGRP) antagonists (9.1%), or monoclonal antibody CGRP antagonists (4.5%) (Table 2).

**Figure 1:**
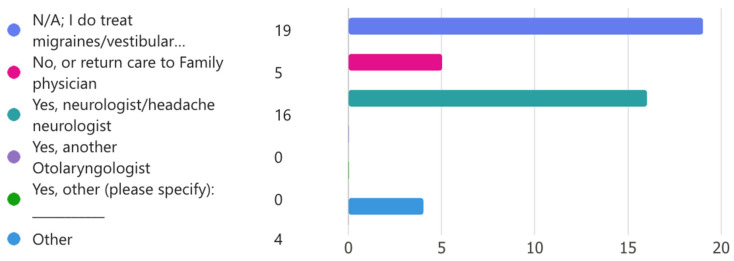
Referral patterns for patients with migraine or vestibular migraine among Canadian otolaryngologists. (See Appendix A for the complete list of survey questions).

When referring patients onward for further treatment, the most common destination was a neurologist (n = 16). A few (n = 5) returned patients to the family physician, while others specified shared care approaches (Figure 1).

**Figure 2:**
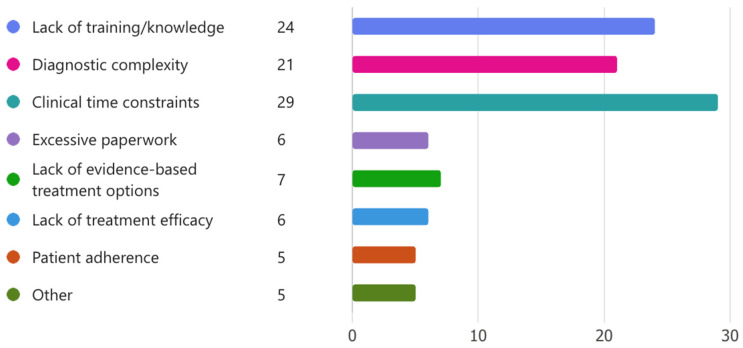
Barriers to VM management.

The most frequently cited barriers to diagnosing and managing VM were clinical time constraints (65.9%), lack of training or knowledge (54.5%), and diagnostic complexity (47.7%) (Figure 2).

**Figure 3:**
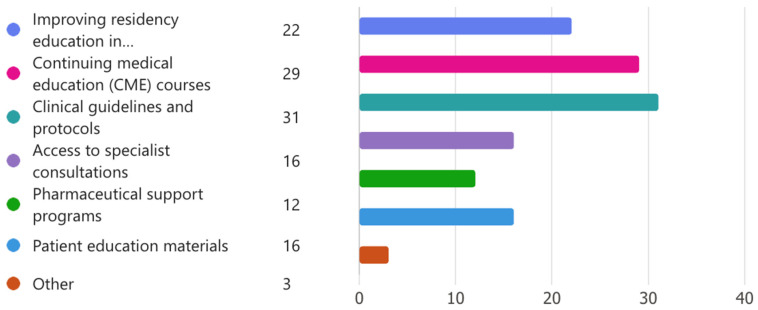
Resources and supports perceived as most helpful for improving the management of vestibular migraine among Canadian otolaryngologists. (See Appendix A for the complete list of survey questions).

Respondents indicated that the most useful resources for improving VM management would include clinical guidelines or protocols (70.5%), continuing medical education (CME) courses (65.9%), improved residency education in migraine/vestibular migraine (50%), access to specialist consultations (36.4%), patient education materials (36.4%), and pharmaceutical support programs (27.3%) (Figure 3).

### Qualitative Feedback

Twelve respondents provided optional free-text comments regarding VM management, with representative examples presented in Table 3. Several recurring themes emerged, including limited access to neurologists with expertise in vestibular migraine, uncertainty regarding specialty ownership of vestibular migraine care, and the substantial time burden associated with counseling and longitudinal management. Multiple respondents described vestibular migraine as a condition requiring multidisciplinary collaboration between otolaryngology, neurology, primary care, and rehabilitation providers. Others highlighted the need for improved patient education materials, easier access to headache-focused resources, and additional continuing medical education opportunities. Some respondents also noted ambiguity between migraine and vestibular migraine terminology and diagnostic categorization in clinical practice.

## 4. Discussion

Among surveyed Canadian otolaryngologists, nearly all respondents reported diagnosing and/or managing VM. Given the low response rate and predominance of otologists and academic practitioners within the respondent cohort, these findings likely reflect the perspectives of clinicians already engaged in dizziness and VM care rather than Canadian otolaryngologists broadly. Nevertheless, the survey provides exploratory insight into current practice patterns, perceived barriers, and educational needs among clinicians involved in VM management.

Despite most respondents reporting familiarity with VM diagnostic criteria, only 15.9% reported extensive residency training specific to migraine or vestibular migraine. This finding highlights a persistent educational gap between the increasing clinical recognition of VM and formal training exposure during residency. Growing academic and clinical interest in vestibular migraine has also been reflected in recent multidisciplinary educational initiatives and conference programming. However, variability in training pathways may contribute to inconsistencies in diagnostic approaches and management practices across providers, consistent with prior literature demonstrating variability in the application of VM diagnostic criteria outside headache-focused specialties [12,13,14].

Lifestyle and dietary modifications, nutraceutical supplementation, tricyclic antidepressants, and analgesics were the most commonly reported management strategies among respondents. These findings generally align with current migraine prevention recommendations and previously published vestibular migraine treatment literature [14,15,16]. Tricyclic antidepressants may represent a familiar treatment option for otolaryngologists given their use in other otolaryngologic conditions, including tinnitus, chronic pain, and laryngeal hypersensitivity disorders [17]. Triptans were also commonly used, suggesting increasing comfort among some otolaryngologists with migraine-directed pharmacotherapy.

Vestibular rehabilitation was used by fewer than one-third of respondents. Although vestibular rehabilitation may benefit selected patients with motion sensitivity, imbalance, or persistent vestibular symptoms, evidence specific to vestibular migraine remains variable, and further study is needed [8,18,19]. Use of CGRP-targeted therapies was also limited among respondents. Although emerging evidence supports the potential benefit of CGRP-targeted therapies in vestibular migraine, these treatments remain largely off-label for VM and may be less familiar to otolaryngologists compared with traditional migraine therapies [15,16,20,21]. Recent studies have reported improvement in vestibular symptom days, headache days, Dizziness Handicap Inventory scores, and vertigo/dizziness attack frequency following rimegepant or anti-CGRP monoclonal antibody therapy in selected VM cohorts, although larger prospective studies are still needed [22,23]. Additional education regarding indications, safety, and evolving evidence may improve clinician familiarity with these newer agents.

The frequency of referrals to neurologists observed in this study highlights the multidisciplinary nature of vestibular migraine management, particularly for patients with refractory symptoms, chronic migraine, post-traumatic migraine, or complex medical comorbidities. At the same time, several respondents noted difficulty accessing neurologists with expertise or interest in vestibular migraine management. Qualitative responses also reflected differing opinions regarding specialty ownership of VM care, with some respondents supporting shared management between otolaryngology, neurology, and primary care providers. These findings underscore the ongoing diagnostic and therapeutic overlap between VM and migraine-related care [9,14]. Recent neuroimaging data further support the concept that VM involves migraine-like functional network alterations, with reported abnormalities in cerebral blood flow and functional connectivity in regions involved in multisensory, autonomic, ocular motor, pain, and emotional processing [24].

Time constraints, diagnostic complexity, and limited training were identified as major barriers to VM management. These findings are consistent with prior literature describing the challenges associated with evaluating dizzy patients and implementing vestibular-focused care pathways [19]. Structured clinical pathways and standardized assessment tools may improve efficiency when evaluating dizzy patients.

Respondents identified clinical guidelines, continuing medical education (CME), and improved residency training as the most valuable resources for improving VM management. Although VM-specific guidelines remain limited, several evidence-based migraine treatment guidelines from Canadian, American, and European headache societies may serve as useful clinical references for otolaryngologists managing these patients [22,25,26,27,28]. Several headache-focused and vestibular-focused CME opportunities are increasingly available to otolaryngologists through national and international educational societies.

Improving education surrounding vestibular migraine at both undergraduate and postgraduate levels may help strengthen diagnostic confidence and management consistency among otolaryngologists [23]. As competency-based medical education frameworks continue to evolve in Canada, greater incorporation of vestibular migraine and dizziness-focused competencies into otolaryngology training may help address current educational gaps [29].

## 5. Limitations

This study has several important limitations. First, the response rate was low (7.4%), introducing substantial risk of response and self-selection bias. Respondents were predominantly clinicians already engaged in dizziness and vestibular migraine care, particularly otologists and practitioners based in academic centers. Consequently, the findings likely reflect the perspectives and management patterns of otolaryngologists with an existing interest in vestibular migraine rather than Canadian otolaryngologists broadly. It does confirm that VM diagnosis and management is seen in the specialty as a sub-specialty area of expertise. Second, the survey instrument was not formally validated or externally piloted, which may have affected question interpretation and response consistency. Since migraine and VM treatment have significant overlap at this time, some questions grouped migraine and vestibular migraine together rather than distinguishing migraine subtypes individually, potentially limiting interpretation of vestibular migraine-specific practices. Third, the modest sample size limited subgroup analyses by subspecialty, years in practice, or practice setting. Geographic underrepresentation from certain provinces and territories further limits national generalizability. Finally, qualitative feedback was limited to a single optional open-ended question with a small number of responses and therefore should be interpreted descriptively rather than as a formal qualitative analysis.

## 6. Conclusions

Among surveyed Canadian otolaryngologists engaged in dizziness and vestibular migraine care, vestibular migraine was commonly encountered and managed using a variety of diagnostic and therapeutic approaches. Considerable heterogeneity remains in training exposure and management strategies, particularly regarding pharmacologic therapies and multidisciplinary care pathways. Respondents identified clinical guidelines, continuing medical education, and improved residency training as key priorities for improving vestibular migraine management. Further efforts toward standardized education and collaborative care models may help improve consistency of care and clinician confidence in managing vestibular migraine.

## Figures and Tables

**Table 1:** Demographic Characteristics of Survey Respondents (n = 44).

Characteristic	Category	Number of Respondents
Province/Territory of Practice	Ontario	13
	Quebec	10
	British Columbia	7
	Alberta	5
	Nova Scotia	4
	Manitoba	2
	New Brunswick	1
	Prince Edward Island	1
	Newfoundland and Labrador	1
	Saskatchewan, Yukon, NWT	0
Primary Practice Focus	Otology/Neuro-Otology	20
	General Otolaryngology	18
	Laryngology	2
	Pediatric Otolaryngology	2
	Rhinology	1
	Head and Neck/Oncology	1
	Facial Plastics/Cosmetics	0
Practice Setting	Academic Teaching Hospital	20
	Community Office/Hospital	14
	Large Non-Academic Hospital	4
	Private Practice Only	3
	Other	3
Years in Practice	0–5 years	8
	6–10 years	12
	11–20 years	13
	21+ years	11
	Retired	0

**Table 2:** Pharmacologic and Non-Pharmacologic Management Strategies Reported by Survey Respondents for Vestibular Migraine.

Treatment Strategy	n (%)
Lifestyle and dietary modifications/trigger avoidance	40 (90.9)
Nutraceuticals and vitamin supplements	26 (59.1)
Tricyclic antidepressants	24 (54.5)
Typical analgesics (e.g., acetaminophen, NSAIDs)	23 (52.3)
Triptans	16 (36.4)
Vestibular rehabilitation therapy/physiotherapy	13 (29.5)
SSRI/SNRI medications	10 (22.7)
Beta blockers	8 (18.2)
Anticonvulsants	8 (18.2)
Betahistine	8 (18.2)
Other antihypertensive medications (e.g., verapamil, candesartan)	5 (11.4)
Gepants	5 (11.4)
CGRP monoclonal antibodies	2 (4.5)
Cognitive-behavioral therapy	2 (4.5)
Vision therapy/neuro-optometric therapies	3 (6.8)
Onabotulinum toxin injections	0 (0.0)

**Table 3:** Representative Themes Identified from Optional Free-Text Responses Regarding Vestibular Migraine Management Among Canadian Otolaryngologists.

Theme	Representative Comment
Limited neurology access	“Neurologists who specialize in headache are a rarity in our city.”
Scope of practice concerns	“VM can be treated by anyone including well educated GPs or general ENTs. It shouldn’t be a referral to neurotology from ENT.”
Time burden	“Diagnosis, counseling, and management of migraine is very time-consuming and not appropriately remunerated.”
Need for multidisciplinary collaboration	“We need better collaboration with neurology, perhaps a joint vestibular migraine meeting between the 2 specialties in Canada.”
Educational resource needs	“A good patient sheet for education, diet, and supplements are a good start for ENT unless they truly have the time and interest to treat further.”

## Data Availability

The original contributions presented in this study are included in the article. Further inquiries can be directed to the corresponding author.

## Abbreviations

The following abbreviations are used in this manuscript:

VM	Vestibular migraine
CSOHNS	Canadian Society of Otolaryngology–Head and Neck Surgery
CME	Continuing medical education
CGRP	Calcitonin gene-related peptide
SSRI	Selective serotonin reuptake inhibitor
SNRI	Serotonin-norepinephrine reuptake inhibitor
CBD	Competency by Design
ICVD	International Classification of Vestibular Disorders
CHS	Canadian Headache Society
AHS	American Headache Society

## Appendix A

### Survey Questions

**No.**	**Survey Question**
1	In which province/territory do you practice?
2	What is your primary practice focus?
3	Where is the majority of your practice based?
4	How many years have you been practicing?
5	How familiar are you with the diagnostic criteria for migraine and vestibular migraine? - Very familiar - Somewhat familiar - Not very familiar - Never heard of it
6	How important do you consider the role of an Otolaryngologist in diagnosing and managing dizzy patients with migraine and vestibular migraine? - Very important - Somewhat important - Neutral - Not so important - Not a part of Otolaryngology
7	Did you receive any formal education specific to migraines and vestibular migraines in your residency? - Yes, extensive training - Yes, some training - No training at all
8	How often do you see and treat dizzy patients in your practice (pick best that applies)? - Multiple times a week - Multiple times a month - Less than once a month - Hardly ever
9	Do you currently diagnose and/or treat patients with migraines and vestibular migraines? - Yes, both diagnose and treat - Yes, only diagnose - No, I do not diagnose or treat these patients
10	If you DO treat patients with migraines or vestibular migraines, what treatments do you commonly use? (Select all that apply) - Lifestyle and dietary modifications and trigger avoidance - Nutraceuticals and vitamin supplements - Adjunct treatments: Anti-nausea medications (e.g., Gravol, metoclopramide) - Adjunct treatments: Betahistine - Abortive treatment: Typical analgesics (e.g., Tylenol, Advil, Naproxen) - Abortive treatment: Devices (e.g., Cefaly, Gammacore) - Abortive treatment: Triptans (e.g., Rizatriptan, Sumatriptan) - Abortive treatment: Gepants (e.g., Ubrogepant, Rimegepant) - Preventive treatments: Tricyclic antidepressants (e.g., nortriptyline, amitriptyline) - Preventive treatments: SSRI/SNRI (e.g., escitalopram, venlafaxine, sertraline) - Preventive treatments: Anticonvulsants (e.g., lamotrigine, valproate, topiramate, gabapentin) - Preventive treatments: Beta blockers (e.g., propranolol, atenolol) - Other blood pressure medications (e.g., flunarizine, verapamil, candesartan) - Calcitonin gene-related peptide (CGRP) blockers - Monoclonal antibodies (e.g., erenumab, fremenezumab) - Onabotulinum toxin injections - Vestibular rehabilitation therapy and other physiotherapy - Vision therapy and other neuro-optometric-based therapies - Cognitive-behavioral therapy - Other
11	If you DO NOT treat migraines or vestibular migraines, do you refer these patients to another specialist? - N/A; I do treat migraines/vestibular migraines - No, or return care to Family physician - Yes, neurologist/headache neurologist - Yes, another Otolaryngologist - Yes, other (please specify) - Other
12	What do you perceive as the main barriers to diagnosing and managing migraines and vestibular migraines in your practice? (Select all that apply) - Lack of training/knowledge - Diagnostic complexity - Clinical time constraints - Excessive paperwork - Lack of evidence-based treatment options - Lack of treatment efficacy - Patient adherence - Other
13	What resources or support would be or would have been most helpful to you in improving your management of migraines and vestibular migraines? (Select all that apply) - Improving residency education in migraine/vestibular migraine - Continuing medical education (CME) courses - Clinical guidelines and protocols - Access to specialist consultations - Pharmaceutical support programs - Patient education materials - Other
14	Do you have any additional comments regarding the management of migraines and vestibular migraines in Otolaryngology practice?
